# The Journey of Mitochondrial Protein Import and the Roadmap to Follow

**DOI:** 10.3390/ijms24032479

**Published:** 2023-01-27

**Authors:** Mary Oluwadamilola Haastrup, Kunwar Somesh Vikramdeo, Seema Singh, Ajay Pratap Singh, Santanu Dasgupta

**Affiliations:** 1 Mitchell Cancer Institute, University of South Alabama, Mobile, AL 36604, USA; 2 Department of Pathology, College of Medicine, University of South Alabama, Mobile, AL 36617, USA; 3 Department of Biochemistry and Molecular Biology, University of South Alabama, Mobile, AL 36688, USA

**Keywords:** mitochondria, mitochondrial protein import machineries, proteins, mitochondrial unfolded protein response, diseases

## Abstract

Mitochondria are double membrane-bound organelles that play critical functions in cells including metabolism, energy production, regulation of intrinsic apoptosis, and maintenance of calcium homeostasis. Mitochondria are fascinatingly equipped with their own genome and machinery for transcribing and translating 13 essential proteins of the oxidative phosphorylation system (OXPHOS). The rest of the proteins (99%) that function in mitochondria in the various pathways described above are nuclear-transcribed and synthesized as precursors in the cytosol. These proteins are imported into the mitochondria by the unique mitochondrial protein import system that consists of seven machineries. Proper functioning of the mitochondrial protein import system is crucial for optimal mitochondrial deliverables, as well as mitochondrial and cellular homeostasis. Impaired mitochondrial protein import leads to proteotoxic stress in both mitochondria and cytosol, inducing mitochondrial unfolded protein response (UPR^mt^). Altered UPR^mt^ is associated with the development of various disease conditions including neurodegenerative and cardiovascular diseases, as well as cancer. This review sheds light on the molecular mechanisms underlying the import of nuclear-encoded mitochondrial proteins, the consequences of defective mitochondrial protein import, and the pathological conditions that arise due to altered UPR^mt^.

## 1. Introduction

Mitochondria are organelles present in almost all eukaryotic cells, and their number per cell depends on their energy demand. Organs with high metabolic activity, for example, heart muscles, kidneys, and the brain, contain the largest number of mitochondria [1,2]. Mitochondria are believed to be the descendants of an ancient prokaryote that underwent an endosymbiotic event with early eukaryotes [3]. Apart from their role in energy production, mitochondria are involved in numerous metabolic processes, including the biosynthesis of amino acids, lipids, heme, and Fe-S clusters [4]. In addition, they also play crucial functions in programmed cell death and maintenance of calcium homeostasis [4,5]. A mitochondrion is a double membrane-bound structure consisting of an outer membrane, intermembrane space (IMS), inner membrane, and matrix [6]. The outer membrane is permeable to solutes up to approximately 5 kDa and characterized by the presence of various enzymes and channels, such as carnitine palmitoyltransferase I, acyl-CoA synthetase, voltage-dependent anion channel (VDAC), and mitochondrial apoptosis-induced channel (MAC) [7]. On the other hand, the inner membrane is impermeable except through specific transporters and contains the enzyme complexes responsible for oxidative phosphorylation [6,8]. The IMS consists of enzymes including caspases, adenylyl kinase, and cytochrome c. Similarly, the mitochondrial matrix consists of several enzymes that take part in metabolic processes such as the tricarboxylic acid cycle and β oxidation of fatty acids. 

**Figure 1:**
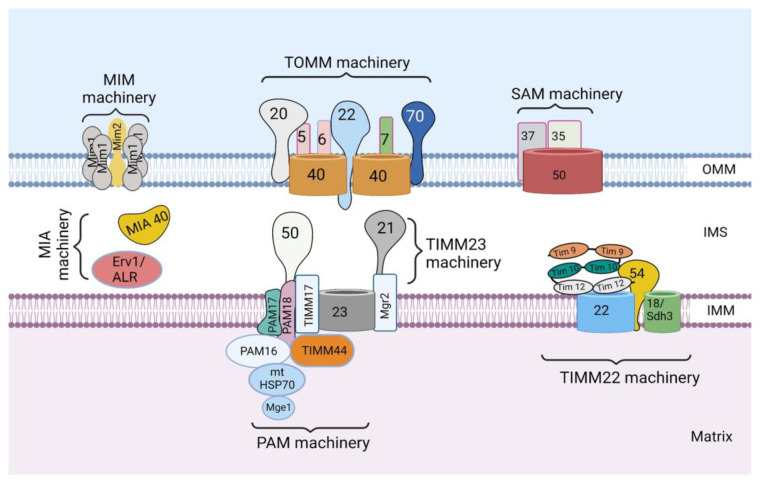
The mitochondrial protein import system. The mitochondrial protein import system consists of seven machineries, including the translocase of the outer mitochondrial membrane (TOMM) machinery, mitochondrial import machinery (MIM), sorting and assembly machinery (SAM), mitochondrial intermembrane space import and assembly machinery (MIA), translocase of the inner mitochondrial membrane 23 (TIMM 23) machinery, translocase of the inner mitochondrial membrane 22 (TIMM 22) machinery, and a presequence-associated motor (PAM). The figure was created with Biorender.com.

Each mitochondrion has its own genome, which is a 16.5 kb double-stranded, closed circular DNA present in the mitochondrial matrix. The mtDNA is strictly maternally inherited and packaged into nucleoids, which is done to ensure its proper distribution and propagation [9]. The mitochondrial genome consists of 37 genes that encode approximately 1% of the total mitochondrial proteins (13 OXPHOS proteins), 2 ribosomal RNAs (12S and 16S rRNA), and 22 transfer RNAs [6]. The remaining 99% of mitochondrial proteins (~1500) are encoded by the nuclear genome, synthesized in the cytosol, and imported into the mitochondria by the mitochondrial protein import system (Figure 1). It was previously believed that all mitochondrial precursor proteins are imported via one main pathway and mechanism [10]; however, further studies revealed the existence of a mitochondrial protein import system comprising several machineries responsible for importing mitochondrial proteins into diverse mitochondrial compartments through various mechanisms specific to each machinery.

Proper functioning of mitochondria hinges on the efficient operation of the mitochondrial protein import system. Hence, impaired mitochondrial protein import leads to certain consequences including defective mitochondrial operations and proteotoxic stress in both the mitochondrial and cytosolic compartments, which are corrected by certain responses including mitochondrial unfolded protein response (UPR^mt^). Altered UPR^mt^ is linked to the development of various pathological conditions including neurodegenerative and cardiovascular diseases, as well as cancer. Here, we review the current understanding of mechanisms underlying the import of nuclear-encoded mitochondrial proteins, the consequences of impaired mitochondrial protein import, and different disease conditions that develop due to altered UPR^mt^.

## 2. Mitochondrial Protein Import System

The mitochondrial protein import system consists of seven machineries: the translocase of the outer mitochondrial membrane (TOMM) machinery, mitochondrial import machinery (MIM), sorting and assembly machinery (SAM), mitochondrial intermembrane space import and assembly machinery (MIA), translocase of the inner mitochondrial membrane 23 (TIMM 23) machinery, translocase of the inner mitochondrial membrane 22 (TIMM 22) machinery, and a presequence-associated motor (PAM) (Figure 1). These machineries are multiprotein complexes composed of protein subunits, which perform specific roles.

The understanding of how the mitochondrial protein import system works is still evolving; however, the TOMM machinery appears to be the most important, as it is the first to come in contact with majority of the nuclear-encoded mitochondrial proteins to allow their entry into the intermembrane space [4]. Subsequently, these proteins make use of any of the other machineries to get to their final destination. The choice of the next machinery depends on the targeting signal and the destination of the protein. MIM promotes the import of signal-anchored and polytopic outer-membrane proteins, whereas SAM is responsible for the insertion of β-barrel proteins in the OMM [4,11]. MIA machinery promotes the import of many intermembrane space proteins. On the other hand, proteins destined for the inner mitochondrial membrane (IMM) are inserted into the IMM by either TIMM23 or TIMM22 machinery [4,11]. Furthermore, matrix proteins are transported into the matrix through the cooperation of TIMM23 and PAM machineries.

## 3. Protein Sorting at the Outer Mitochondrial Membrane

The mitochondrial outer membrane possesses two types of integral membrane proteins, including β-barrel proteins that are integrated into the OMM by multiple transmembrane β strands, and α-helical proteins, which are anchored in the OMM by one or more hydrophobic α-helical segments [4,10]. 

### 3.1. Sorting of β-Barrel Proteins into the Outer Mitochondrial Membrane

The presence of β-barrel proteins in the OMM is a key feature of the outer membrane of Gram-negative bacteria, reflecting the origin of mitochondria from prokaryotes [12]. Examples of these proteins are voltage-dependent anion channel (VDAC), TOMM40, and SAM50 [4]. The first machinery involved in the import of these β-barrel precursors is the TOMM machinery, which is composed of four receptors—TOMM20, TOMM70, and two molecules of TOMM22, as well as two molecules of the transmembrane channel—TOMM40, and three small subunits—TOMM5, TOMM6, and TOMM7—which are essential for complex stability and assembly [4,13]. However, it is unknown whether both TOMM40 channels take part in the translocation of incoming precursors across the OMM. In addition, how the two TOMM22 receptors cooperate during the recognition and binding of the incoming precursors remains to be elucidated. 

**Figure 2:**
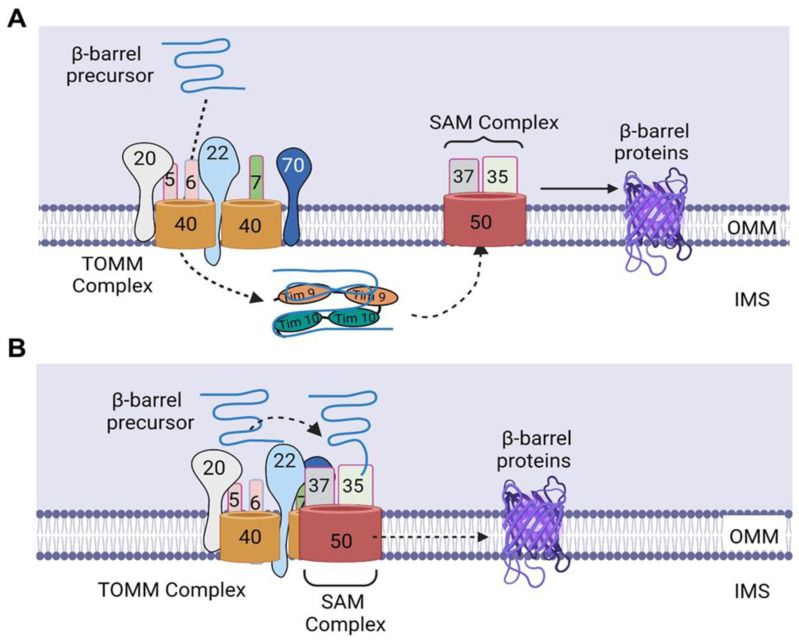
Import of β-barrel precursors into the outer mitochondrial membrane. (**A**) Upon translocation through the TOMM40 channel, the precursors bind to TIMM9–TIMM10 complex, which protect the β-barrel precursors from aggregation in the aqueous IMS and deliver the β-barrel precursors to the SAM complex. Subsequently, the precursors are folded in the SAM complex and laterally released into the lipid phase of the outer membrane. (**B**) SAM37 interacts with the cytosolic receptor domain of TOMM22, thereby linking the two complexes and leading to the formation of a TOMM–SAM supercomplex, which enables the binding of SAM35 to the β signal of the precursor, thereby allowing the direct transfer of the β-barrel precursors from TOMM to the SAM complex. Subsequently, the β-barrel precursors are inserted into the SAM50 channel, after which they are folded in the SAM complex and released laterally into the lipid phase of the outer membrane. IMS, intermembrane space. The figure was created with Biorender.com.

After synthesis in the cytosol, the β-barrel precursors are recognized by the TOMM receptors and guided through the TOMM40 channel, through which they enter the IMS [12]. The identification of these β-barrel precursors by the TOMM receptors is directed by a targeting signal that consists of a β-hairpin element containing two adjacent β-strands, which are the two most C-terminal β strands of the precursor, and the connecting loop [4,14]. Although the exact sequence of the recognition of the targeting signal by the TOMM receptors is not completely understood, TOMM20, 70, and 22 have been shown to be crucial for the import of β barrel precursors [15,16]. Upon translocation through the TOMM40 channel, the precursors are bound to the small TIMM chaperones of the IMS, which exist as heterohexameric complexes, including the TIMM9–TIMM10 and TIMM8–TIMM13 complexes [4,12]. Of these, the TIMM9–TIMM10 complexes have been identified to be the main form involved in transfer of many hydrophobic proteins through the IMS [4,17]. These chaperones protect the β-barrel precursors from aggregation in the aqueous IMS and deliver the β-barrel precursors to the SAM complex (Figure 2A) [4,12], which consists of a membrane-integrated protein, SAM50, and two peripheral membrane proteins exposed to the cytosol, SAM35 and SAM37 [4]. The exact mechanisms governing the insertion of β-barrel proteins into the OMM are still not completely understood; however, it is believed that the SAM complex is responsible for the membrane insertion of these proteins (Figure 2A). 

In contrast to the understanding that the β-barrel proteins are imported first into the IMS before being transferred to the SAM complex, Kutik et al. proposed that translocation of β-barrel precursors into the SAM complex is initiated by the binding of the last β-strand (β signal) to SAM35, a SAM subunit located on the cytosolic surface of mitochondria [18,19]. This signal binding then induces a conformational change that leads to opening of the SAM50 channel; thus, several β strands can be inserted into a hydrophilic, proteinaceous membrane environment [18]. Subsequently, the precursors are folded in the SAM complex and laterally released into the lipid phase of the outer membrane [18,19]. The binding of this β signal to a SAM subunit not integrated into the lipid phase of the outer membrane but embedded into a proteinaceous membrane environment by its close association with SAM50 molecules [18] suggests that the β-barrel precursors are not imported into the IMS but transferred directly from the TOMM complex to the SAM complex (Figure 2B).

In addition to the SAM components—SAM50, SAM35, and SAM37—which are required for β-barrel formation, TOMM22 has been shown to be required for β-barrel folding, as the oxidation of TOMM40, a β-barrel protein, was observed to be impaired in mitochondria lacking TOMM22 [15]. The promotion of β-barrel folding by TOMM22 could be due to its interaction with a fraction of SAM subunits, connecting the TOMM and SAM complexes and resulting in the generation of a TOMM–SAM supercomplex [15], suggesting that the formation of the supercomplex is essential for the folding of the β barrel. In another study by Wenz et al., it was shown that SAM37 is the sole SAM subunit responsible for the formation of the TOMM–SAM supercomplex, as deletion of *SAM37* blocked the copurification of other SAM subunits with TOMM22_His_ (His-tagged TOMM22) [20]. In addition, overexpression of SAM35 did not restore the interaction of TOMM and SAM complexes in *SAM37*Δ mitochondria [20]. Furthermore, the authors showed that SAM37 interacts with the cytosolic receptor domain of TOMM22, linking the two complexes and leading to the formation of a TOMM–SAM supercomplex (Figure 2B) [20]. The identification of this supercomplex supports the possibility of β-barrel precursors being transferred directly from TOMM to the SAM complex, as the two complexes are brought close to each other through the formation of the supercomplex, thereby enabling the binding of SAM35 to the β signal and the subsequent insertion of the β barrel precursors in the SAM50 channel. Therefore, further mechanistic studies are needed to determine whether this direct transfer is attainable, as well as to uncover novel insights about the step-by-step mechanisms involved in the import of nuclear-encoded β-barrel mitochondrial proteins.

### 3.2. Sorting of α-Helical Proteins into the Outer Mitochondrial Membrane

The mechanisms involved in the import of α-helical proteins is only partly understood. Three classes of α-helical proteins have been identified, including signal-anchored proteins, tail-anchored proteins, and polytopic (multispanning) outer-membrane proteins [4]. Signal-anchored and tail-anchored proteins contain an α-helical transmembrane segment at the N terminus and C terminus, respectively, which function as both membrane anchors and targeting signals, in addition to flanking positively charged amino acid residues [4]. In contrast, polytopic proteins possess multiple transmembrane segments that may contain targeting information; however, their exact targeting signals are unknown. 

**Figure 3:**
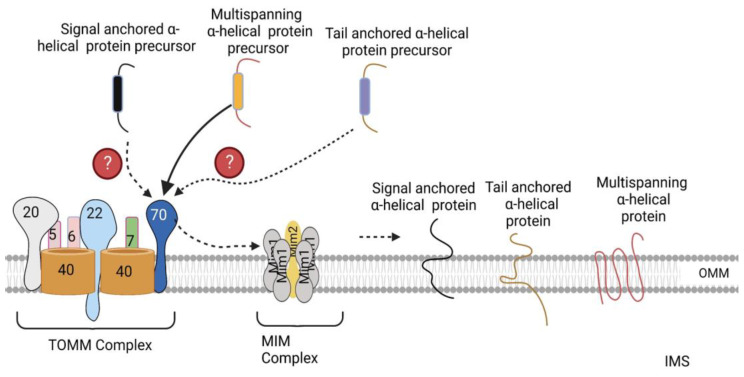
Import of α-helical precursors into the outer mitochondrial membrane. Polytopic proteins are recognized by the TOMM70 receptor, after which TOMM70 binds to them and transfers them to the MIM complex, which inserts them into the OMM. Signal- and tail-anchored α-helical precursors are also inserted into the OMM by the MIM complex. The exact TOMM receptors recognizing these precursors have not been identified yet. OMM, outer mitochondrial membrane. The figure was created with Biorender.com.

The MIM complex consisting of multiple copies of Mim1 and one or two copies of Mim2 has been identified as the machinery promoting the insertion of signal-anchored and polytopic α-helical proteins into the OMM (Figure 3) [4]. However, the molecular mechanisms through which it inserts these proteins into the OMM has not been elucidated yet [4]. No TOMM receptor has been identified to date that is required for the import of signal-anchored proteins [21,22]. In contrast, TOMM70 has been observed to recognize the precursors of polytopic proteins, after which, it binds them and transfers them to the MIM complex, which inserts them into the OMM (Figure 3) [23,24]. Previously, no proteinaceous machinery had been identified for the import of tail-anchored α-helical proteins; however, a recent study by Doan et al. showed that the import of the radiolabeled precursor of Fis1, a tail-anchored α-helical protein, into isolated *mim1*Δ and *mim1-23* mitochondria was impaired [25]. In addition, re-expression of Mim1 in *mim1*Δ yeast mitochondria promoted the import of Fis1 [25]. Thus, the MIM complex is essential for the import of all three α-helical proteins. Besides the MIM complex, the low ergosterol content of the outer membrane favors the insertion of tail-anchored proteins into the OMM [26]. Likewise, the precursor of Ugo1, a polytopic protein, has been shown to be inserted into protein-free liposomes that mimic the phospholipid composition of the OMM [27], suggesting that the lipid composition of the mitochondria play a role in the optimal insertion of α-helical proteins. 

Some OMM proteins are inserted into the outer membrane via routes distinct from those mentioned above. For example, Mcp3, an OMM protein, contains an N-terminal presequence and a stop-transfer signal, which allows it to be imported by the TOMM and TIMM23 machineries, after which it is released laterally into the inner membrane, where it is processed by the inner-membrane peptidase (IMP). Subsequently, it is released into the IMS and exported into the outer membrane by the MIM complex [28]. This suggests that other import machineries, apart from the import machineries located on the OMM, are involved in the import of OMM proteins. Therefore, more studies are needed to identify additional machineries involved in the import of OMM proteins and the proteins imported by these machineries. Furthermore, because the import of α-helical proteins is only understood in part, more studies with the aim of achieving an improved understanding of the mechanisms underlying the import of these proteins are needed. 

## 4. Protein Sorting into the Intermembrane Space

**Figure 4:**
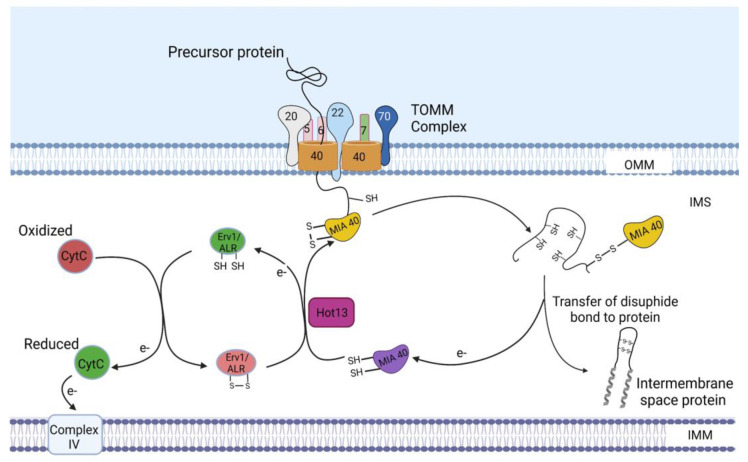
Import of intermembrane space proteins. As the precursors pass through the TOMM40 channel, the IMS sorting signals of these precursors are recognized by MIA40. Thereafter, MIA40 binds the precursors and facilitates their entry into the IMS. The imported precursors are oxidized by the oxidoreductase activity of MIA40, after which they are assembled in the IMS. In turn, MIA40 becomes reduced and is reoxidized by Erv1/ALR with the assistance of the zinc-binding protein–Helper of Tim protein 13 (Hot13). Electrons derived from the oxidation of the imported precursors by MIA40 are transferred to Erv1/ALR and, subsequently, to cytochrome C and complex IV. IMS, intermembrane space; Erv1, essential for respiration and viability 1 protein; ALR, augmenter of liver regeneration. The figure was created with Biorender.com.

Many mitochondrial intermembrane space proteins contain characteristic cysteine motifs, such as Cx_3_C and Cx_9_C motifs, that form intramolecular disulfide bonds [4,29]. These proteins are kept in a reduced state in the cytosol after synthesis and translocated through the TOMM40 channel across the outer membrane [30,31]; however, none of the TOMM receptors was found to be a requirement for these proteins [31,32]. After emerging on the IMS surface of the TOMM40 channel, the IMS sorting signal of these precursors consisting of a hydrophobic element flanked by a cysteine residue is recognized by mitochondrial intermembrane space import and assembly protein 40 (MIA40), a component of the MIA machinery (Figure 4) [33]. Thereafter, MIA40 binds the precursors and facilitates their entry into the IMS, functioning as an IMS receptor [34]. In addition to aiding in the translocation of precursors into the IMS, binding of MIA40 to the precursors leads to the formation of a disulfide bond between MIA40 and the cysteine of the mitochondrial intermembrane space sorting signal [4], which is later transferred to the imported precursor (Figure 4). The transfer of disulfide bonds to the precursors leads to their oxidation and promotes their conformational stabilization and assembly in the IMS [4]. 

As these precursors become oxidized, electrons are transferred to MIA40, leading to its reduction. MIA40 then becomes reoxidized by essential for respiration and viability 1 protein/Augmenter of liver regeneration (Erv1/ALR), the second component of the MIA machinery. In addition to Erv1/ALR, the zinc-binding protein-Helper of Tim protein 13 (Hot13) promotes the oxidation of MIA40 by keeping it in a zinc-free state [35]. Oxidation of MIA40 by Erv1 leads to the transfer of a disulfide bond to MIA40, which serves as the source of the disulfide bond being transferred from MIA40 to its substrates. During this process of disulfide bond transfer from Erv1 to MIA40, electrons transferred from the oxidized substrates to MIA40 are transferred to Erv1, which then passes the electrons to cytochrome C and, subsequently, to complex IV (Figure 4) [36,37]. 

**Figure 5:**
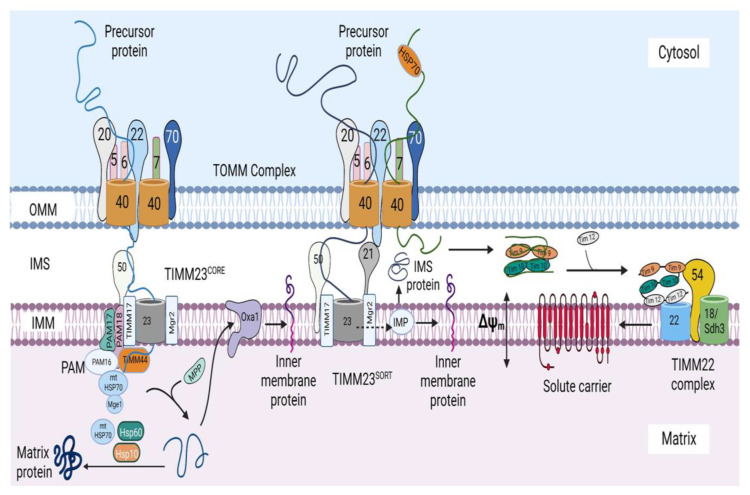
Import of inner-mitochondrial membrane and matrix proteins. The presequence-carrying precursors are first recognized by TOMM20, which binds to them and transfers them to TOMM22, after which they are translocated through the TOMM40 channel, through which they enter the IMS, where they bind to the IMS domain of TOMM22. TIMM21 then binds the IMS domain of TOMM22, thereby promoting the dissociation of the precursors. Thereafter, TIMM50 binds to the precursor proteins and transfers the precursors into the TIMM23 channel. The Δψm exerts an electrophoretic effect on the positively charged N terminus of precursors and activates the TIMM23 channel, thereby aiding in the movement of the precursors through the TIMM23 channel. The hydrophobic sorting signals of the precursors are then recognized by Mgr2, which then binds to the sorting signals and controls the release of the precursors into the inner membrane. Subsequently, the inner-membrane peptidase (IMP) removes the hydrophobic sorting sequences, and the mature proteins are either released into the IMS or remain anchored in the inner membrane by an additional hydrophobic segment. Precursor proteins containing presequences devoid of hydrophobic sorting signals are destined for the matrix and are imported through the cooperation of the TOMM, TIMM23^CORE^, and PAM machineries. After translocation through the TOMM40 channel, these precursors bind to the IMS domain of TOMM22, after which TIMM50 binds these precursors and transfers them to the TIMM23 channel. TIMM44 then binds to the precursor as it emerges on the matrix side of the TIMM23 channel and transfers it to mtHsp70, which imports the protein into the matrix. The presequences are removed by the matrix processing peptidase (MMP), and the proteins are folded into their mature forms by the soluble form of mtHSP70 and the HSP60-HSP10 chaperonin complex. Oxa1 aids in the export of some of the transmembrane segments of some inner-membrane precursors from the matrix into the inner mitochondrial membrane. Following their synthesis in the cytosol, carrier precursors are bound to cytosolic chaperones of the Hsp70 and Hsp90 classes to prevent aggregation. Thereafter, these chaperones deliver the precursors to TOMM70, which then transfers this precursor to TOMM22, after which they are transferred to the TOMM40 channel and translocated across the OMM in a loop formation. The small TIM chaperones of the IMS are recruited to the channel exit by an N-terminal segment of the channel protein TOMM40. TIMM54, a subunit of the TIMM22 machinery, recruits the small TIMM chaperones to the TIMM22 complex, after which, the precursors are delivered to the TIMM22 channel. The Δψm activates the TIMM22 channel and exerts an electrophoretic effect on the carrier precursors, which aids in the movement of the precursors through the channel. Finally, the precursors are released laterally from the TIMM22 complex into the IMM. OMM, outer mitochondrial membrane; IMM, inner mitochondrial membrane, IMS, intermembrane space; Δψm, mitochondrial membrane potential. The figure was created with Biorender.com.

Apart from the MIA machinery, some IMS proteins can be imported by the TOMM/TIMM23^SORT^ machineries (see Section 5) (Figure 5). Furthermore, some matrix and inner-membrane proteins are substrates of the MIA machinery. For example, the precursor of TIMM17, a subunit of the TIMM23 machinery, interacts with the hydrophobic binding pocket of MIA40, which facilitates its import [4,38]; however, the exact signal for recognition by MIA40 was not identified. Additionally, TIMM17 was found to be directly oxidized by Erv1, leading to the formation of a disulfide bond, which is critical for efficient protein translocation through the TIMM23 complex and dynamic gating of the TIMM23 channel [38]. The direct oxidation of TIMM17 by Erv1 raises the possibility that Erv1 may directly interact with a number of substrates of the MIA machinery [4,38], leading to the formation of disulfide bonds independent of the oxidoreductase activity of MIA40.

## 5. Protein Sorting at the Inner Mitochondrial Membrane and Matrix

Precursor proteins destined for the IMM contain a cleavable presequence at their N terminus, which serves as their targeting signal. Additionally, they contain a hydrophobic sorting signal, also known as a stop-transfer sequence [4]. However, not all precursors destined for the IMM contain presequences or the stop-transfer sequence. The carrier precursors are the second group of proteins destined for the IMM and contain internal targeting elements, which function as their targeting signals [4]. Both groups of IMM proteins are imported by the TOMM machinery into the IMS; however, their import into the IMM involves two different IMM-localized machineries. The precursors containing presequences are imported into the IMM by the TIMM23 machinery, whereas those containing internal targeting elements are imported into the IMM by the TIMM22 machinery [39]. Precursors destined for the matrix are synthesized with only cleavable presequences and are imported into the matrix through the cooperation of the TOMM, TIMM23, and PAM machineries [4,39].

### 5.1. Import of Precursors Containing Presequences 

An important characteristic of mitochondrial presequences is the formation of an amphipathic α-helix that contains a positively charged face and a hydrophobic face [40]. TOMM20 forms the initial receptor for each presequence. It specifically recognizes and binds the hydrophobic surface of the amphipathic helix [41], after which the presequence is handed over to TOMM22, which binds to the positively charged surface. Subsequently, the presequence is transferred to TOMM40, through which it enters the IMS, where it binds to the IMS domain of TOMM22 [42,43] (Figure 5). 

Two different forms of TIMM23 machinery have been described for the import of presequence-carrying precursors [12,44] known as TIMM23^SORT^ and TIMM23^CORE^ (Figure 5). The TIMM23^SORT^ machinery is responsible for the import of presequences with a hydrophobic sorting signal into the IMM, whereas the TIMM23^CORE^ machinery is involved in the import of presequence-carrying proteins devoid of hydrophobic sorting signals into the matrix.

#### 5.1.1. Sorting of Precursors Containing Presequences into the Inner Mitochondrial Membrane

The TIMM23^SORT^ machinery is composed of five subunits: TIMM21, TIMM17, TIMM50, TIMM23, and Mgr2. TIMM21 is known to bind to the IMS domain of TOMM22, thereby promoting the dissociation of the precursor, as well as bringing the TOMM and TIMM23^SORT^ machineries in close contact [44,45]. Thereafter, TIMM50 binds to the precursor protein, after which the precursor is transferred to the TIMM23 channel, a subunit of the TIMM23 machinery, through which precursor proteins enter the IMM and the matrix. TIMM50 is also responsible for regulating the gating of the TIMM23 channel. Although the underlying mechanism is not well understood, Meinecke et al. demonstrated that TIMM50 induces the closure of the TIMM23 channel to prevent ion leakage and dissipation of mitochondrial membrane potential (Δψm) in the absence of precursors, whereas upon binding of a presequence, the channel is activated and opened [46], allowing precursor proteins to pass through. Similarly, TIMM17, a subunit known to be closely associated with TIMM23, has also been reported to regulate the gating of the TIMM23 channel [38]. 

Once the precursor proteins are in the TIMM23 channel, the hydrophobic sorting signal is recognized by Mgr2, which then binds to the sorting signal and controls the release of the precursor into the inner membrane [4]. Afterwards, the inner-membrane peptidase (IMP) removes the hydrophobic sorting sequence, and the mature protein is either released into the intermembrane space or remains anchored in the inner membrane by an additional hydrophobic segment (Figure 5) [4]. Thus, the TIMM23^SORT^ complex promotes the import of precursors containing both presequences and hydrophobic sorting signals destined for the IMM or IMS. However, it is presently unclear how the presequences of these precursors are removed.

#### 5.1.2. Sorting of Precursors Containing Presequences into the Matrix

In contrast to the TIMM23^SORT^ machinery, the TIMM23^CORE^ machinery lacks a TIMM21 subunit [44]. Upon translocation of precursors containing only cleavable presequences through the TOMM40 channel, these precursors bind to the IMS domain of TOMM22 [42,43]. Subsequently, TIMM50 binds these precursors and transfers them to the TIMM23 channel. 

The PAM machinery composed of an ATP-driven molecular chaperone, mitochondrial heat-shock protein 70 (mtHsp70), TIMM44, PAM16, PAM17, PAM18, and nucleotide exchange factor Mge1, is responsible for completing the import of these precursors into the matrix (Figure 5) [4]. It is anchored to the TIMM23^CORE^ machinery via the interaction between the N-terminal domain of PAM18 and TIMM17 (Figure 5). As the precursor emerges on the matrix side of the TIMM23 channel, TIMM44 binds to it and transfers it to mtHsp70, which tightly binds the unfolded polypeptide chain with its peptide-binding domain [4]. Its ATP-hydrolyzing activity is regulated by PAM18 (also known as TIMM14) and PAM16 (TIMM16), whereas Mge1 promotes the release of ADP from it to initiate a new round of the reaction cycle [4]. Although the mechanism through which the PAM machinery imports the precursors into the matrix is not completely understood, two models have been proposed, known as the trapping and pulling models. In the trapping/Brownian ratchet model, binding of mtHsp70 to the precursor in transit prevents backsliding of the polypeptide chain through the import channels, and thus, through Brownian motion, the polypeptide moves inward until another mtHsp70 molecule binds to it. The precursor is thus imported in a stepwise manner into the matrix by ATP-dependent cycles of mtHsp70 binding and release [4]. In contrast to this model, in the pulling model, the import motor plays a more active role. MtHsp70 binds to TIMM44 and interacts with the precursor in transit. Conformational changes of mtHsp70 during its ATPase cycle generate an inwardly directed force on the precursor. Afterwards, another mtHsp70 binds to the polypeptide chain, pulling it further into the matrix [4]. 

After the import of these proteins into the matrix, their presequences are removed by the matrix processing peptidase (MPP). In addition, other proteases, such as the octapeptidyl aminopeptidase and the intermediate cleaving peptidase (Icp55), remove destabilizing N-terminal amino acid residues from the imported proteins [4,47]. Subsequently, the soluble form of mtHSP70 and other chaperones, for example, the HSP60-HSP10 chaperonin complex, promote folding of the proteins into active forms [39,48]. 

Although it is understood that TIMM21 dissociates from the TIMM23^SORT^ machinery in order for the TIMM23^CORE^ machinery to associate with the PAM machinery for the import of matrix proteins [44], the exact mechanism underlying the decision is made to switch from a TIMM23^SORT^ to a TIMM23^CORE^ complex and vice versa is unknown. Thus, studies aimed at understanding how this decision is made are needed. It was suggested by van der Lann et al. that the interaction between TIMM21 and the IMS domain of TOMM22 is regulated by the import signals of incoming precursors [49]. Therefore, it is possible that TIMM21 is able to recognize the presence of a hydrophobic sorting signal in presequences, which triggers its binding to the IMS domain of TOMM22, thereby promoting the dissociation of the precursor from TOMM22. On the other hand, in the absence of a hydrophobic sorting signal in the presequence-carrying precursor, TIMM21 dissociates from the TIMM23^SORT^ complex [44], resulting in the formation of the TIMM23^CORE^ machinery. 

Apart from the subunits of the TIMM23 machinery, the Δψm also plays critical roles in the translocation of precursors across the inner membrane by exerting an electrophoretic effect on the positively charged N terminus of precursors and activating the TIMM23 channel [50,51]. Furthermore, TIMM21 has been shown to link the TIMM23^SORT^ complex to the respiratory chain III–IV supercomplex (*bc*_1_ complex and cytochrome *c* oxidase (COX)), thereby bringing the TIMM23^SORT^ complex in close proximity to proton-pumping complexes, where the electrochemical proton gradient is higher, which, in turn, promotes precursor translocation [52]. 

Some inner-membrane precursors are imported into the inner membrane by a combination of stop-transfer and conservative sorting mechanisms [4]. In the conservative sorting route, precursors are imported by TOMM, TIMM23^CORE^, and PAM machineries into the matrix, followed by export into the inner membrane via Oxa1, the main component of the OXA machinery (Figure 5) [4]. Examples of such precursors imported via the combination of stop-transfer and conservative sorting mechanisms are multispanning inner-membrane precursors. The ABC transporter Mdl1, a multispanning inner-membrane protein, contains six α-helical transmembrane segments, of which two middle transmembrane segments are imported into the matrix in a PAM-dependent manner and inserted into the inner membrane by Oxa1, whereas the other transmembrane segments are sorted by a stop-transfer mechanism [53]. Similarly, TIMM18 and Sdh3, subunits of the TIMM22 machinery are cleavable multispanning inner-membrane proteins, which have also been shown to be imported by a combination of conservative sorting (the first two transmembrane segments) and stop transfer (the third transmembrane segment) [54]. 

### 5.2. Import of Carrier Precursors into the Inner Mitochondrial Membrane 

Carrier precursors are synthesized without presequences but contain several internal targeting elements distributed over the mature primary structure, which function as targeting signals [4,10]. Examples include ADP/ATP carrier and phosphate carrier. After synthesis in the cytosol, the carrier precursors are bound to cytosolic chaperones of the Hsp70 and Hsp90 classes to prevent aggregation [4]. Thereafter, these chaperones deliver the precursors to TOMM70 [55], which possesses binding sites for both the precursors and chaperones [56]. TOMM70 then transfers this precursor to TOMM22, after which it is inserted into the TOMM40 channel. 

In contrast to cleavable precursors, which are translocated across the OMM in a linear manner, carrier precursors are inserted into the TOMM40 channel in a loop formation such that both termini are still on the cytosolic side, whereas the middle portion of the precursor passes through the channel [57]. The N-terminal segment of TOMM40 that passes from the cytosolic side through the interior of the β-barrel channel into the intermembrane space then recruits small TIMM chaperones of the IMS to the channel exit such that the hydrophobic precursors can be directly transferred to the chaperones as they enter the IMS [4]. As stated above (in Section 3), the main form of the small TIMM chaperone complex is the soluble TIMM9–TIMM10 complex. After binding the precursors, another small TIMM protein, TIMM12, associates with the TIMM9–TIMM10 complex, thereby forming the TIMM9–TIMM10–TIMM12 hexameric chaperone complex [12,58]. Subsequently, TIMM54, a subunit of the TIMM22 machinery, recruits the TIMM9–TIMM10–TIMM12 complex to the outer surface of the TIMM22 complex, where it binds the complex [59], thereby delivering the precursors to the TIMM22 channel, another subunit of the TIMM22 machinery, which mediates the insertion of precursor proteins into the inner membrane (Figure 5). Although the mechanism through which carrier precursors are inserted into the IMM is not completely understood, Rehling et al. proposed that Δψm promotes the opening of the TIMM22 channel in the presence of a carrier signal peptide and exerts an electrophoretic force on the incoming precursor [60], thereby aiding the movement of the precursor into the TIMM22 channel, after which it is laterally released from the TIMM22 complex into the IMM, where it assembles into its mature, functional form [12]. 

Apart from TIMM54, TIMM22, and the TIMM9–TIMM0–TIMM12 complex, other subunits of TIMM22 machinery include TIMM18 and Sdh3, which together form a TIMM18–Sdh3 module, the second pore of the TIMM22 complex [4]. Although, their exact roles in the import of carrier proteins are unknown, it is believed that they play a role in the assembly of the TIMM22 complex [4,61]. TIMM18 and Sdh3 are evolutionarily related to the membrane-integral part of respiratory complex II (succinate dehydrogenase, SDH) of mitochondria and bacteria [4]. While TIMM18 shows similarity to Sdh4, Sdh3 possesses a dual localization as a subunit of both respiratory complex II and of the TIMM22 complex [61]. However, it is unknown whether the dual localization of Sdh3 leads to a functional cross talk between the respiratory chain and carrier translocase [4]. Therefore, further studies are needed to understand whether a functional cross talk occurs between these two complexes. Furthermore, additional investigations are required with the aim of elucidating the exact roles played by Sdh3 and TIMM18 during the import of carrier precursors, as well as the exact mechanisms involved in the insertion of carrier precursors into the IMM.

In addition to carrier precursors that contain six transmembrane segments [62], the TIMM22 machinery also imports other non-cleavable proteins with only four transmembrane segments into the IMM, including TIMM17, TIMM22, and TIMM23 [12].

## 6. Consequences of Defective Mitochondrial Protein Import

Besides cytosolic proteostasis, mitochondrial proteostasis is also vital for cellular stress resistance and organismal health [63]. Therefore, proteostasis in these compartments needs to be maintained for proper cellular functioning. Defective mitochondrial protein import leads to reduced levels of nuclear-encoded mitochondrial proteins and impaired mitochondrial functioning, as well as proteotoxic stress, which triggers a mitochondrial-to-nuclear signal transduction pathway called mitochondrial unfolded protein response (UPR^mt^) [64,65]. This pathway reacts to proteotoxic stress in mitochondria by increasing the expression of mitochondrial chaperones, such as mitochondrial presequence translocase-associated motor complex protein (mtDNAj), heat shock 10 kDa protein (Hsp10), and heat shock 60 kDa protein (Hsp60), as well as proteases such as ATP-dependent Clp protease proteolytic subunit (ClpP), Lon protease homolog, mitochondrial (LONP1), YME1-like 1 ATPase, (YME1L), and OMA1 zinc metallopeptidase (OMA1) [66,67], thereby promoting the refolding of misfolded proteins, as well as degradation of damaged mitochondrial proteins. In addition, impaired mitochondrial protein import results in the accumulation of mitochondrial precursor proteins in the cytosol, which leads to proteotoxic stress, also called mitochondrial precursor overaccumulation stress (mPOS) [68,69]. Cells are equipped with several tiers of responses to alleviate such stress and restore homeostasis [68,70,71,72]. These responses include increased expression of cytoplasmic chaperones, attenuation of translation, and increased proteasome activity via the upregulation of proteasome expression and the promotion of proteasome assembly, which supports the clearance of mislocalized precursors from the cytosol [66,68,71].

When these protective mechanisms occurring in the mitochondria and cytosol become defective, the accumulated misfolded proteins in the mitochondria, as well as the unimported mitochondrial precursors in the cytosol, form aggregates [66,73], which lead to the development of diseases such as neurodegenerative and heart diseases, among many others [66]. Although UPR^mt^ activation is a promising therapeutic option for many conditions, its overactivation could lead to undesired side effects, such as cancer development [74,75]. Similarly, the overactivation of cytosolic responses to proteotoxic stress including overexpression of cytosolic chaperones and excessive proteasome activity promotes cancer development and progression [76,77,78,79]. The following section of this review focuses on the pathological conditions that occur due to altered UPR^mt^.

### 6.1. Pathobiological Implications of Altered Mitochondrial Unfolded Protein Response

#### 6.1.1. Parkinson’s Disease 

Parkinson’s disease (PD) is the second most prevalent neurodegenerative disorder, affecting 1–3% of the population over 65 years of age [80], with most cases being sporadic and approximately 10–15% of patients with a family history of the disease [6]. Clinical features include tremors, akinesia or bradykinesia, progressive rigidity, and postural instability [6]. The neuropathological hallmarks of this pathology include loss of dopaminergic neurons in the midbrain substantia nigra pars compacta (SNpc) and accumulation of Lewy bodies (LBs) containing α-synuclein [80]. Although the molecular mechanisms underlying this neurodegeneration are still not fully understood, various studies have shown that impaired mitochondrial functioning (i.e., mitochondrial dysfunction) and abnormal protein aggregation are two of the major contributors to PD [81]. As mentioned earlier, proper functioning of the mitochondrial protein import system is crucial for optimal mitochondrial functioning, as defective mitochondrial protein import leads to reduced bioenergetic capacity and increased oxidative stress, thereby contributing to the development of PD [82,83]. This reduction in mitochondrial protein import also leads to accumulation of misfolded proteins in the mitochondria, resulting in proteotoxic stress, which activates UPR^mt^. Although it is not clear how defective UPR^mt^ leads to Parkinson’s disease, studies have shown that the induction of UPR^mt^ protects against neurodegeneration [84]. In addition, activation of UPRmt promotes dopamine neuronal survival and longevity in Parkinson’s disease animal models [85]. Similarly, the inhibition of UPRmt results in decreased lifespan and dopamine neuronal loss [85].

Because UPR^mt^ is essential for the folding of misfolded proteins and the degradation of damaged proteins, one possible mechanism through which its inhibition can promote the development of PD is that inhibition of UPR^mt^ leads to increased levels of misfolded mitochondrial proteins, which are dysfunctional, resulting in defective mitochondrial respiration and enhanced production of ROS that contribute to the induction of neuronal cell death [86]. Furthermore, impaired UPR^mt^ can lead to aggregation of misfolded proteins, thereby causing further impairment in mitochondrial function. For example, the aggregated form of α-synuclein has been shown to inhibit mitochondrial complex I, leading to reduced respiration and ATP production in neurons, in addition to an increase in ROS production, resulting in neuronal death [87]. Interestingly, this aggregated α-synuclein is the main component of the Lewy bodies in PD [88]. Despite various pieces of evidence showing mitochondrial localization of α-synuclein and interaction with components of the electron transport chain [87,89,90], a study by Wang et al. suggested that pathogenic α-synuclein is not present inside mitochondria but is membrane-bound and associated with these organelles. In support of this theory, Di Maio suggested that there is no specific interaction between monomeric α-synuclein and the TOMM machinery; however, the oligomeric forms of α-synuclein bound to TOMM20, which prevented the interaction of TOMM20 with TOMM22, thereby resulting in impaired mitochondrial import of endogenous or exogenous presequence-containing, nuclear-encoded proteins, which, in turn, led to deficient mitochondrial respiration, enhanced production of ROS and loss of MMP [91]. Activation of UPR^mt^ in response to decreased mitochondrial protein import helps to improve mitochondrial function [92]. On the other hand, the impairment of UPR^mt^ in this case would lead to increased mitochondrial stress and dysfunction, resulting in increased neuronal death. 

Conversely, overactivation of UPR^mt^ has been suggested to increase the accumulation of defective mitochondria [93]. Furthermore, Martinez et al. showed that overactivation of UPR^mt^ leads to increased neurotoxicity of α-synuclein, which promotes the death of dopaminergic neurons in a nonapoptotic manner [93]. This suggests that the degree of activation of UPR^mt^ determines its effect in PD. Therefore, further studies are needed to confirm the exact roles of UPR^mt^ in the pathogenesis of PD and how optimal UPR^mt^ can be maintained; this would help to achieve an improved understanding of how mitochondrial dysfunction leads to the development of PD and provide novel insights about the development of mitochondria-targeted therapies for better management of PD patients.

#### 6.1.2. Alzheimer’s Disease

Alzheimer’s disease (AD) is the most common form of dementia in the elderly population. Clinically, AD is defined by cognitive impairment that is pervasive enough to interfere with a person’s ability to work or complete daily activities [75]. AD can be divided into different types based on age of onset and genetic predisposition. Sporadic or late-onset AD accounts for more than 95% of cases and begins after the age of 65 years. Early-onset or familial AD is rare and usually manifests by age 60 [94]. The main neuropathology features of AD include extracellular amyloid plaques consisting of polymers of amyloid-β peptides (Aβ) and intracellular neurofibrillary tangles formed mainly by hyperphosphorylated protein tau [95]. Although the exact cause of AD is unknown, genetic evidence predicts that oligomeric species of Aβ are the likely cause [96]. Evidence has also shown that mitochondrial dysfunction plays a key role in AD pathogenesis, as proper mitochondrial functioning is essential for neuronal health [97,98]. 

Pathologies of AD such as the deposition of amyloid β and tau are known to result in impairments in mitochondrial function [99]. This could occur through the inhibition of mitochondrial protein import. Devi et al. showed that Alzheimer’s amyloid precursor protein 695 (APP), a plasma membrane protein known to be the source of the toxic amyloid β (Aβ) peptide associated with the pathogenesis of Alzheimer’s disease (AD), formed a stable ~480 kDa complex with the TOMM40 import channel in mitochondria of human AD brains but not in age-matched controls, thereby inhibiting mitochondrial protein import, which led to defective mitochondrial functioning [100]. Aβ has been reported to localize to the mitochondrial matrix, where it interacts with the mitochondrial matrix protein, amyloid-beta-binding alcohol dehydrogenase (ABAD), leading to mitochondrial dysfunction and increased ROS generation [101]. Furthermore, pathological tau impairs mitochondrial dynamics by regulating mitochondrial fission/fusion proteins, resulting in mitochondrial dysfunction [102]. In addition, abnormal tau disturbs mitochondrial bioenergetics by inhibiting complex 1 activity, decreasing Δψm and ATP levels, and increasing ROS production [103]. 

The defect in mitochondrial protein import occurring as a result of APP clogging the TOMM40 channel could lead to an increase in misfolded proteins in the mitochondria, resulting in the activation of UPR^mt^. Likewise, oxidative stress occurring as a result of excessive ROS generation disrupts the protein folding mechanism, thereby enhancing the production of misfolded proteins [104], which, in turn, results in UPR^mt^ activation. Although the role of UPR^mt^ in AD pathogenesis is not yet completely understood, most studies show that UPR^mt^ activation plays a protective role in AD. Sorrentino et al. showed that inhibition of UPR^mt^ aggravates the disease. Conversely, boosting mitochondrial proteostasis by increasing UPR^mt^ decreased protein aggregation and delayed disease progression [105]. Another study showed that Honokiol, a small-molecule polyphenol isolated from the bark of *Magnolia officinalis*, activated UPR^mt^, which improved cognitive impairment in AD mouse models [106]. In addition, UPR^mt^ is strongly activated and exerts a protective role against Aβ protein toxicity in PITRM1-knockout iPSC-derived cortical neurons and cerebral organoids. In line with this, pharmacological inhibition of UPR^mt^ exacerbates Aβ proteotoxicity in cerebral organoids generated from PITRM1-knockout iPSCs [107]. Thus, these studies indicate that defective UPR^mt^ promotes AD progression.

Although no studies have been conducted to date on the effect of UPR^mt^ overactivation on AD pathogenesis, Beck et al. showed that compared to control subjects, the expression of UPR^mt^-related genes was increased by 40–60% in sporadic AD subjects and 70–90% in familial AD subjects [108]. The activation of these genes in the postmortem frontal cortex samples of these subjects indicate that this physiologically important cellular response may be chronically activated in AD, perhaps as a compensatory neuroprotective response to a sustained accumulation of unfolded, misfolded, and damaged mitochondrial proteins [108]. Thus, further studies are needed to examine the consequences of persistent UPR^mt^ activation. Furthermore, additional studies are needed with the aim of determining the time period during which UPR^mt^ becomes activated in disease development. Such studies would help to provide guidance about the development of UPRmt-based therapeutics for AD management.

#### 6.1.3. Cardiovascular Diseases

Cardiovascular diseases, especially acute myocardial infarction (MI) and chronic heart failure (HF), account for numerous deaths and severely undermine quality of life [109]. Mitochondrial dysfunction has been identified as a crucial etiological factor for these diseases by contributing to energetic dysfunction, oxidative stress, calcium dysregulation, and cardiomyocyte death and is considered a potential therapeutic target [109,110]. 

A crucial stress response triggered by mitochondrial dysfunction is UPR^mt^, which has been shown to play cardioprotective roles in cardiovascular diseases [111]. Venkatesh et al. showed that upregulation of LONP1 mitigates cardiac injury by preventing oxidative damage of proteins and lipids, preserving mitochondrial redox balance, and reprogramming bioenergetics by reducing Complex I content and activity [112]. On the other hand, LONP1 activity, which is reduced by oxidative stress, leads to the accumulation of dysfunctional respiratory chain subunits and left ventricle contractile dysfunction [113], indicating that defective UPR^mt^ promotes the development of cardiovascular diseases. In another study by Smyrnias et al., it was demonstrated that UPR^mt^ was induced in the hearts of mice subjected to chronic hemodynamic overload [67]. Additionally, boosting UPR^mt^ with nicotinamide riboside reduced cardiomyocyte death and contractile dysfunction in mice subjected to pressure overload [67]. A study by Wang et al. also confirmed that pharmacological UPR^mt^ activation exerts cardioprotective effects in an activating transcription factor 5 (ATF5)-dependent manner in mouse models of ischemia–reperfusion injury [111]. Moreover, myocardial tissue from patients with aortic stenosis also showed evidence of UPR^mt^ activation, which correlated with reduced tissue cardiomyocyte death and fibrosis, as well as lower plasma levels of biomarkers of cardiac damage (high-sensitivity troponin T) and dysfunction (N-terminal pro-B-type natriuretic peptide) [67], supporting a protective role of UPRmt in pathological heart conditions. Conversely, enhanced sympathoexcitation leads to decreased UPR^mt^, resulting in increased proteotoxic stress, which results in decreased OXPHOS and Δψm as well as increased ROS generation, which, in turn, induces mitochondrial transition pore opening, activating pro hypertrophy/fibrosis factors that induce pathological cardiac hypertrophy and fibrosis [114]. These findings suggest that although UPR^mt^ is cardioprotective, its activation status varies in different heart conditions. Therefore, further studies are required to determine the status of UPR^mt^ activation in different heart conditions and the period during which this response becomes activated in the development of the disease.

On the other hand, some studies have shown that overactivation of UPR^mt^ promotes the progression of heart diseases. Liu et al. showed that elevated activity of Omi/HtrA2 protease promotes mitochondrial depolarization and apoptosis in aged rat heart [115]. Likewise, in vitro and in vivo overexpression of mitochondrial Omi/HtrA2 induces cardiac apoptosis and dysfunction [116]. A potential explanation for these contradictory findings is that UPR^mt^ is cardioprotective when moderately active, whereas its excessive activity may be cardiotoxic [109]. A moderate activation of UPR^mt^ may be beneficial for removing/repairing damaged mitochondrial proteins, thereby maintaining optimal mitochondrial and cardiac function, whereas excessive UPR^mt^ activation could result in a massive cleavage of mitochondrial proteins, exacerbating mitochondrial dysfunction and promoting heart damage [109]. Thus, further studies are needed to determine the causes of UPRmt overactivation and how a balance can be properly maintained.

#### 6.1.4. Cancer

The initiation and development of cancer is a multistep process that involves the acquisition of diverse functions, such as resistance to apoptotic cell death, prevention of growth inhibition, and activation of proliferation signals [117]. Cancer cells experience mitochondrial stress as they undergo unchecked cellular proliferation and generate ROS, which damage mtDNA and mitochondrial proteins, including components of the OXPHOS family, causing mitochondrial dysfunction [118]. Cancer cells rely on functional mitochondria to generate macromolecules, such as amino acids, nucleotides, and cholesterol, to maintain their high proliferative capacity. Thus, cancer cells activate the mitochondrial stress response to alleviate mitochondrial dysfunction and protein aggregation, which subsequently promotes tumor growth and progression [118].

Numerous studies have validated these notions by showing that activation of the UPR^mt^ is indispensable for cancer development and progression. In recent studies, Chen et al. showed that UPR^mt^ components (Hsp10, Hsp60, and ClpP) are abundantly expressed in breast cancer tissues compared to adjacent noncancerous tissues [119]. In addition, Kenny et al. demonstrated that persistent activation of UPR^mt^ provides survival advantage to cancer cells, leading to tumor progression [120]. Moreover, high expression of UPR^mt^-related genes is significantly associated with poor clinical outcomes [120]. In the following section, we discuss the roles of key UPR^mt^ components in cancer. 

ATF5, a key UPR^mt^ regulator in mammals, has been observed to be upregulated in glioblastoma, breast, pancreas, rectal, and ovarian cancers [121,122,123,124,125]. In addition, high ATF5 expression correlates with reduced survival in glioma and lung cancer patients [126,127]. Besides promoting cancer growth via recovery of mitochondrial functions, ATF5 has also been shown to promote proliferation and survival of glioma and breast cancer cells by regulating Egr-1 expression [128]. Similarly, Dluzen et al. demonstrated that ATF5 promotes survival of glioblastoma and breast cancer cells by transactivating Bcl2 [129]. 

CIpP, a key protease involved in UPR^mt^, is known to be upregulated in breast cancer, prostate cancer, and acute myeloid leukemia [130,131,132]. Knockdown of ClpP is associated with reduced proliferation, migration, and invasion of breast cancer cells [130]. Additionally, ClpP silencing was reported to lead to decreased oxidative phosphorylation and mitochondrial metabolism, as well as viability of AML cells [132]. Moreover, xenograft studies demonstrate that ClpP knockdown suppresses the growth of prostate-cancer-derived liver metastases [131]. In contrast, overexpression of ClpP increases migratory and invasive activity in breast cancer [130].

LONP1, another key protease involved in UPR^mt^, is upregulated in various cancers, including melanoma, colorectal cancer, and pancreatic cancer [133,134]. Its increased expression has also been reported to correlate with reduced overall survival in neuroblastoma, breast cancer, renal cell carcinoma, and colorectal cancer [135]. LONP1 silencing leads to reduced proliferation in melanoma, colorectal cancer, and pancreatic cancer [133,134]. In addition, a deficiency in LONP1 expression in mice was reported to inhibit the formation and growth of azoxymethane and dextran sulfate-induced colorectal tumors [133]. This could be due to the fact that loss of LONP1 inhibits the formation of OXPHOS complexes I and III, thereby impairing normal mitochondrial functioning [133], which is required for the growth and development of tumors. In another study, silencing of LONP1 in prostate cancer cells resulted in the accumulation of misfolded subunits of OXPHOS complexes II and V; reduced assembly of OXPHOS complexes I, III, IV, and V; and inhibition of activities of OXPHOS complexes I, II, and V [135], resulting in impaired oxidative bioenergetics and heightened ROS production. This, in turn, suppressed mitochondrial trafficking to the cortical cytoskeleton, shut off tumor cell migration and invasion, and inhibited primary and metastatic tumor growth in vivo [135], indicating that defective UPR^mt^ inhibits tumor growth and progression. 

Although not much is known about the role of HSP10 in cancer, evidence has shown that it is overexpressed in multiple forms of cancer, including astrocytoma, oral squamous cell carcinoma, and nasopharyngeal carcinoma [136,137,138]. In addition, increased HSP10 expression is associated with reduced overall survival in astrocytoma, oral squamous cell carcinoma, and nasopharyngeal carcinoma [136,137,138]. Fan et al. showed that increased expression of HSP10 promoted survival and tumorigenesis via the inhibition of apoptosis [136].

HSP60, another key component of the UPR^mt^, is overexpressed in many cancer types, and its expression has been correlated with the metastatic potential of cancers and overall survival of cancer patients [139,140,141,142]. HSP60 knockdown leads to severe deficiencies in mitochondrial functions, which hinder cell growth and survival [143]. Specifically, in pancreatic cancer, HSP60 knockdown reduces the expression of subunits in OXPHOS complexes I, III, IV, and V; disrupts the formation of complexes I and III; and blocks mitochondrial respiration and ATP production [144], indicating that increased activation of HSP60 promotes optimal mitochondrial functioning, which, in turn, promotes tumor growth and progression. In addition to direct mitochondrial functions, HSP60 regulates the expression and release of IL-8 in prostate and colon cancers, possibly via transforming growth factor-beta (TGF-*β*), to enhance cell survival [145]. Besides its established localization in the mitochondria, HSP60 also localizes to other cellular compartments, such as the cytosol, plasma membrane, and extracellular space of cancer cells [118,143]. Cytosolic HSP60 interacts with the IKK complex and enhances activation of IKK [146], resulting in the increased expression of NF- ΚB targets and promotion of cell survival [146]. Interestingly, HSP60 is not overexpressed in all cancers and is not necessarily associated with a poor prognosis in certain patients [143]. For example, HSP60 is downregulated in bronchial cancer, colorectal cancer, and hepatocellular carcinoma [147,148,149]. Furthermore, overexpression of HSP60 suppresses cell proliferation in clear cell renal cell carcinoma and inhibits invasive activity in hepatocellular carcinoma [149,150]. This suggests that HSP60 has multimodal functions in diverse cancers. In addition, because HSP60 is a key component of UPR^mt^, these findings suggest that increased UPR^mt^ activation might have diverse roles in different cancers. Therefore, more studies are needed to investigate whether UPR^mt^ activation has tumor-inhibitory roles. 

## 7. Conclusions

Highly orchestrated governance and legitimate activity of the mitochondrial protein import system are pivotal for both mitochondrial and cellular homeostasis. At present, knowledge about how precisely the mitochondrial protein import system operates is still maturing. Thus, further in-depth studies are warranted to decipher the structures of these machineries, as well as a complete understanding of the various mechanisms involved in the import, sorting, and assembly of mitochondria-targeted proteins. From the above discussion, it is clear that defective mitochondrial protein import could lead to the generation of proteotoxic stress in both mitochondrial and cytosolic compartments. Cells try to combat these stresses via specific responses, including UPR^mt^ and other compensatory mechanisms. Alterations in these responses result in the development of various disease conditions. On the other hand, the abundant activity of the proteins of these machineries (TOMM, TIMM, SAM, etc.) may lead to sustained functions of the mitochondrial energy metabolism and other relevant pathways that are beneficial for aggressive tumor growth and metastasis.

Whereas most studies report that increased activation of UPR^mt^ plays a protective role in neurodegenerative and heart diseases, few studies show that overactivation of UPR^mt^ promotes the progression of these diseases. In contrast to the findings observed in neurodegenerative and cardiovascular diseases, most studies have demonstrated that increased UPR^mt^ activation is essential for cancer development and progression, whereas few studies suggest that increased expression of UPR^mt^ components inhibits cancer progression. Therefore, further studies are needed with the aim of determining the exact roles of UPR^mt^ in various disease conditions and identifying the relevant mechanisms through which alterations in UPR^mt^ result in the development of pathological conditions. The understanding gained from such studies could aid in directing the development of UPR^mt^-based therapies for the management of various pathological conditions.

## Data Availability

Not applicable.
